# Development and external validation of an admission risk prediction model after treatment from early intervention in psychosis services

**DOI:** 10.1038/s41398-020-01172-y

**Published:** 2021-01-11

**Authors:** Stephen Puntis, Daniel Whiting, Sofia Pappa, Belinda Lennox

**Affiliations:** 1 Department of Psychiatry, University of Oxford, Warneford Hospital, Oxford, UK; 2 West London NHS Trust, London, UK; 3 Department of Psychiatry, Imperial College London, London, UK

**Keywords:** Schizophrenia, Prognostic markers

## Abstract

Early Intervention in psychosis (EIP) teams are the gold standard treatment for first-episode psychosis (FEP). EIP is time-limited and clinicians are required to make difficult aftercare decisions that require weighing up individuals’ wishes for treatment, risk of relapse, and health service capacity. Reliable decision-making tools could assist with appropriate resource allocation and better care. We aimed to develop and externally validate a readmission risk tool for application at the point of EIP discharge. All persons from EIP caseloads in two NHS Trusts were eligible for the study. We excluded those who moved out of the area or were only seen for assessment. We developed a model to predict the risk of hospital admission within a year of ending EIP treatment in one Trust and externally validated it in another. There were *n* = 831 participants in the development dataset and *n* = 1393 in the external validation dataset, with 79 (9.5%) and 162 (11.6%) admissions to inpatient hospital, respectively. Discrimination was AUC = 0.76 (95% CI 0.75; 0.77) in the development dataset and AUC = 0.70 (95% CI 0.66; 0.75) in the external dataset. Calibration plots in external validation suggested an underestimation of risk in the lower predicted probabilities and slight overestimation at predicted probabilities in the 0.1–0.2 range (calibration slope = 0.86, 95% CI 0.68; 1.05). Recalibration improved performance at lower predicted probabilities but underestimated risk at the highest range of predicted probabilities (calibration slope = 1.00, 95% CI 0.79; 1.21). We showed that a tool for predicting admission risk using routine data has good performance and could assist clinical decision-making. Refinement of the model, testing its implementation and further external validation are needed.

## Introduction

The impact of schizophrenia and related psychotic disorders on an individuals’ quality of life, health, and social functioning can be debilitating and life-limiting^1,2^. These illnesses are large contributors to the global burden of disease^3^. Recent systematic reviews are more optimistic on the outcomes of first-episode psychosis (FEP) and with early treatment, many people will reach both remission and recovery^4^. Early intervention in psychosis (EIP) services are community mental health teams that provide the gold standard for treatment of FEP^5^. They aim to reduce the likelihood of a poor illness trajectory by shortening the duration of untreated psychosis and providing a comprehensive and assertive treatment regime early in the course of the illness. However, these type of interventions are time-limited, with individuals treated for between 2 and 3 years dependent on health service and region. At the end of EIP, individuals are either discharged by their clinician to a primary care provider or transferred to a standard adult community mental health team.

### Clinical decision making

To make a decision about the correct post-discharge care, a treating clinician will have to take into consideration the individual’s wishes for further treatment, their likely need for further intensive mental health treatment, and the current availability and capacity of secondary care services^6–8^. In addition, the next care provider may often have its own eligibility criteria or diverging opinions about the need for continued specialist mental health input^9,10^. In health systems without universal health coverage, these decisions become even more difficult, as availability of primary care services may not be guaranteed, or access may be based on or limited by, appropriate medical insurance.

Discharge decisions are not straightforward and without risk. Hospital readmissions in the 2 years after EIP treatment are relatively common (~17.9% within 2 years). This is reduced for those discharged to primary care (12.1%) in comparison to those transferred to community mental health teams (35%), but within these 2 years, more than 30% of individuals discharged to primary care will be referred back to community mental health services^11,12^. While some of these relapses and readmissions may be due to reasons beyond the control of mental health services, they may contribute to this risk through underestimation of the individuals’ need for specialist mental health care, leading to an individual’s deterioration and eventual relapse. Both clinicians^6,7^ and individuals themselves^8^ share the concern that those who have maintained good mental health during EIP with its specialist, multidisciplinary support, can relapse after EIP treatment has ended. Transition periods in psychiatry (and throughout medicine) are historically and habitually a point of high risk^13,14^. Good practice discharge guidelines for EIP exist^15^, but the eligibility criteria for access to treatment can differ by region and by service capacity at the time of discharge^6^.

### Applying prediction models to clinical decision making

One approach to improve discharge decision making is to provide decision tools to clinical staff at the point of care^16^. Decision tools are routinely used in medicine. They are used to target preventative treatment at those most likely to benefit, for example, the QRISK tool is used in cardiovascular disease for primary prevention^17^. They are also commonly used to help inform discussions around complex decisions, for example, in cancer treatment^18,19^. Decision tools can also be used to help in decisions around resource allocation, such as the Ottawa Ankle Rules for decisions around using X-rays for possible fractures^20^.

Psychiatric risk-assessment tools are common, but risk prediction is less common, in particular for psychosis. Most of the substantive psychosis risk prediction literature aims to predict the transition from people in ‘at-risk’ states to a psychotic illness^21,22^. Many of these suffer from poor methodology, with a few exceptions that have been externally validated and are being tested in clinical services^23^. We are aware of one machine learning algorithm developed to predict recovery following the first episode of psychosis^24^. Properly developed and validated prediction of the risk of readmission to hospital is rare^25^.

Computer-assisted guidance on decision-making can improve patient therapeutic outcomes^26^. An important, if not the most important aim of clinical prediction models is to positively affect clinical practice, yet most models are never validated nor implemented in clinical services^27,28^. Barriers to adoption of these models include poor model transportability or lack of effectiveness on outcomes, a lack of acceptability for patients and clinicians, and lack of usability due to non-automation within existing electronic health records (EHRs) or poor presentation of the model results^27–29^. Pragmatic, easily useable tools with little burden on the clinician are more easily implemented in standard clinical practice^27,30^.

With this in mind, we aimed to develop a prognostic tool to help clinical decision making around the risk of readmission at the end of EIP treatment. We developed our prognostic tool with its implementation in mind, selecting predictors that are routinely collected and already available as structured within electronic health records (EHRs), aiming to maximise clinical feasibility and reduce clinician burden by allowing automation and incorporation within the EHR user interface for EIP services. We timed our prognostic tool measurement to coincide with a pre-determined medical review prior to the transfer of care for the move from EIP services to the next care provider. Finally, our prognosis tool focuses on a clinical decision with an outcome that is associated with considerable uncertainty, has significant implications for both individuals and primary and secondary care services, and has been highlighted by clinicians and people with psychosis as an area of clinical need^6^.

This study is the first to investigate whether routinely recorded data can be used to develop and validate a risk prediction tool for relapse requiring admission to a psychiatric inpatient hospital within 12 months of discharge from EIP services.

## Methods

### Design

We used retrospective cohort data from electronic health records (EHRs) for both development and validation datasets.

### Data sources

For both development and validation data, we used the UK Clinical Record Interactive Search Tool (UK-CRIS) to access the Oxford Health NHS Foundation Trust (the development cohort) and West London NHS Trust (the external validation cohort) EHR clinical registers. UK-CRIS is a platform that provides a technological and governance model so that researchers are able to access pseudonymised medical records held in mental health NHS Trusts. The use of UK-CRIS for anonymised secondary data retrieval has been approved by the National Health Service Health Research Authority (HRA) and therefore does not require individual study ethical approval. All UK-CRIS projects in both Oxford Health NHS Foundation Trust and West London NHS Trust are submitted to independent CRIS Oversight Groups within each region for approval.

Oxford Health NHS Foundation Trust is the primary provider of both inpatient and outpatient mental health care in the counties of Oxfordshire and Buckinghamshire, England, serving a population of 1.2 million. The counties have both rural and urban areas with mostly lower deprivation than the national average, although Oxford city has pockets of very high deprivation (amongst the 20% most deprived in England). Oxford Health NHS Foundation Trust has two EIP teams covering the Oxfordshire and Buckinghamshire areas, respectively, with average annual caseload sizes of ~250 and 130, respectively.

West London NHS Trust provides care and treatment for children, adults and older people living in the London boroughs of Ealing, Hammersmith & Fulham and Hounslow, delivering services in the community (at home, in GP surgeries, care homes), hospital specialist clinics and forensic (secure) units, serving a population of ~1 million. The three boroughs are urban and very diverse both in terms of ethnicity and deprivation index. There are three borough-based EIP services Hammersmith and Fulham, Hounslow, and Ealing with caseload sizes of ~140, 150 and 200, respectively.

There is a higher prevalence of psychosis in West London in comparison to Oxford and Buckinghamshire, with crude incidence rates between 16 and 31 per 100,000 person-years in Oxfordshire and Buckinghamshire in comparison to between 31 and 43 per 100,000 person-years in boroughs of West London^31^.

EIP services in both NHS trusts did not go through major structural changes during the data collection period, although both were subject to the introduction of an NHS waiting time standard in 2016 that required that more than 50% of all referrals to EIP services commence a package of treatment within 14 days of referral.

### Model development

Our eligible sample was all individuals aged between 14 and 65 who were referred to Oxford Health NHS Foundation Trust EIP services between 1st January 2011 and 8th October 2019 (Supplementary Fig. 1). Individuals were not part of the study population if they were still under the care of EIP at the end of study date or if they had <12 months of follow-up since their discharge from EIP services at the end of study date as we were interested in 1-year post-EIP discharge outcomes. Individuals were excluded if there was no EHR data held for the individual if they had moved out of area, if they never received an assessment or contact from the EIP team following their initial referral, or if they only received an EIP assessment and were deemed not eligible for EIP treatment. Individuals were also excluded if they remained a psychiatric hospital inpatient throughout their period of EIP care. Although these participants would have been assigned to the caseload of EIP services, this is to provide a link to future community care after discharge from hospital, as EIP services are exclusively community services. In practice, the inpatient team would have had clinical responsibility for all treatment and discharge decisions during the period where these participants were on the caseload of the EIP team, and this is therefore not a relevant population in terms of the target decision for the model.

Follow-up started at the end-of-treatment date for EIP services and ended 1 year on from that date. Our primary outcome was admission to an inpatient psychiatric unit within 12 months of the end of EIP treatment. We chose a psychiatric admission as we considered it to have: face validity, in that it captures a clear deterioration in mental state; utility, as it has clear relevance to resource use and service planning; and accuracy, given that NHS Trusts are required by the national authorities to record inpatient bed use meaning few administrative errors or incomplete data. We only considered the first and not subsequent admissions. We chose a 12-month follow-up as our previous study identified that the majority of readmissions occur within 12 months of discharge from the service^11^.

We derived the development model with logistic regression. We adjusted the model for age at discharge from the EIP service, gender, ethnicity, quintile of social deprivation, diagnosis prior to discharge, duration of EIP care (in days), the number of previous admissions to a psychiatric hospital at discharge, and having a diagnosis of a substance misuse disorder. We measured social deprivation from the neighbourhood-level Index of Multiple Deprivation. The IMD combines seven domains (income deprivation; employment deprivation, education skill and training development, health deprivation and disability, crime, barriers to housing and services, and living environmental deprivation) to give an overall deprivation score for 32,844 distinct geographical areas in England.

We pre-selected our candidate predictors based on previous literature of sociodemographic and clinical characteristics associated with relapse following discharge from EIP services^11^ and consultations with clinical teams, clinical academics and NHS Trust informatics staff about routinely collected and reported data in EIP services. Our primary aim was to embed a clinical focus to the tool from the start of the process and so we did not select candidate predictors that were not routinely collected in clinical services. We aim to provide the tool as automatic decision support as part of clinical workflow and therefore wanted to reduce any additional data entry need^30^. We included demographic variables of age, gender, and ethnicity in our model despite them being non-significant in our previous study^11^ due to the considerable contrary evidence that these are important factors in the outcomes of people with psychosis^32^. Discharge destination was not kept in the model as the aim of this model is to aid clinical decision making in discharge decisions and therefore the tool would be used prior to the individual’s discharge destination being known. We dichotomised categorical variables with more than two categories to reduce overfitting of parameters included in the model, but we did not dichotomise continuous measures^33^. The choice to use this reduced number of parameters was based on our previous study where we obtained an estimate for our development sample of the number of participants who were likely to be eligible and the number of admissions^11^. In total, we included 8 predictor parameters—age, gender, ethnicity (white or non-white), LSOA, diagnosis (schizophrenia or non-schizophrenia), number of days under EIP care, number of previous admissions to a psychiatric hospital and a substance misuse diagnosis, all of which remained in the final model. We prioritised a diagnosis of schizophrenia over other diagnoses in our reduced parameter model as in this instance it has higher face validity and incidence in this cohort.

We imputed missing data via multiple imputations using chained equations. We used the predictive mean matching method for imputation of numerical data and logistic regression for binary data^34^. We produced 20 imputations and our estimates for the final model were obtained by pooling across imputations according to Rubin’s Rules^35^.

We derived regression coefficients from the fitted logistic model. We assessed the internal validity of the development model using bootstrapping, creating 250 samples drawn with replacement from the development dataset^33^. We performed bootstrap validation on each imputed dataset prior to pooling. We assessed overall model performance by calculating the Brier score, which is the mean squared difference between the predicted probability assigned to the possible outcomes for an item and the actual outcome of that item. Brier scores range between 0 and 0.25, with lower scores indicating better performance^36^. We assessed discrimination by calculating the concordance-index^33^. Discrimination is a representation of the ability of a prediction model to correctly categorise individuals who did or did not experience the event of interest^37^. The concordance-index is scored between 0–1, with 1 representing perfect discrimination and 0.5 representing no better than chance.

We also assessed the calibration of the model. Calibration is an indicator of the level of agreement between the observed outcome and the predicted probabilities of that outcome in the model^37^. Calibration is often ignored in the development of clinical prediction models but is arguably more important than discrimination—as poorly calibrated models will give systematically off-target predictions which would not be clinically useful^28^. We assessed calibration through calibration-in-the-large, the calibration slope and calibration plots. Calibration-in-the-large compares the mean of all predicted risks with the mean observed risk and gives an indication of whether predictions are systematically too high (with scores below 0) or too low (with scores above 0). The calibration slope is the slope of the line of agreement between expected and observed outcomes, with a slope of 1 and an intercept of 0 (i.e. a prediction line on the 45-degree line) suggesting perfect agreement between observed and expected outcomes.

We conducted a sensitivity analysis to compare the imputed model performance to model performance of complete cases only.

### External validation

Our eligible validation sample was all individuals aged between 14 and 65 who were referred to West London NHS Trust services between 31st January 2006 and 18th June 2019 (Supplementary Fig. 1). Exclusion criteria were the same as the development sample. We imputed missing data as in the development sample, resulting in 20 imputed datasets. All analyses were conducted on individual imputations and the resulting statistics were combined using Rubin’s rules^35^.

For the first stage of our model validation, we compared baseline sociodemographic and clinical characteristics of the development and validation samples to look at differences in case-mix.

We then applied the regression coefficients derived from the development model to each individual in the validation dataset and calculated their predicted probabilities. From these probabilities, we calculated discrimination using the concordance-index and estimated overall model performance with the Brier score. We assessed calibration with calibration-in-the-large, the regression slope, and calibration plots of the 20 imputed datasets.

#### Recalibration

We did not re-estimate the validation model by developing a new regression model from the validation sample data. Rather, we updated the intercept and rescaled coefficients using the intercept and calibration slope as an overall adjustment factor and recalculated discrimination and calibration statistics and plots^37^.

We used R version 3.5.0 for all cleaning and analysis of data^38^. Code is available on request. We conducted this study in accordance with Transparent Reporting of a multivariable prediction model for Individual Prognosis Or Diagnosis (TRIPOD) guidelines (Supplementary Table 1^39^).

We further explored the clinical usefulness of the model in comparison to standard clinical decision making using decision curve analysis in our validation dataset^40^. Decision curve analysis quantifies the net-benefit of introducing a test or decision tool by plotting a range of thresholds for which an individual would likely be designated as a ‘case’ (i.e. admitted to a psychiatric hospital) by the test. It is the difference between the proportion of true positives and false positives weighed by the odds of the selected threshold. The model with the higher net-benefit at any one threshold is considered the better model. We compared our prediction model against an individual’s actual discharge destination (primary versus secondary care) to ascertain the net-benefit of using our model over standard clinical discharge outcomes in the prediction of admission within a year of leaving EIP. While there are a number of fixed and time-varying factors that may influence readmission besides the clinical decision of discharging an individual to primary care or transferring them to further secondary care, we considered this decision as to the most pragmatic comparison for our prediction model.

## Results

### Sample characteristics of the development and validation cohorts

We identified 2563 individuals accessing Oxford Health NHS Foundation Trust EIP services between 1st January 2011 and 8th October 2019. 1086 were either still under EIP treatment or did not have 12 months of follow-up since discharge. After removing individuals who met exclusion criteria there were 831 eligible individuals in the development dataset (Supplementary Fig. 1). For external validation, we identified 3212 individuals accessing West London NHS Trust EIP services between 31st January and 18th June 2019. After removing those still with the EIP team or without 12 months follow-up (*n* = 986) and the individuals who met exclusion criteria there were 1393 eligible individuals remaining.

Table 1 outlines the demographic and clinical characteristics of both samples. Individuals had an average age of 25.6 years (SD = 7.6) in the development and 26.7 years (SD = 5.2) in the external validation datasets, respectively, with those in West London older. 37% were female in both datasets. There were more individuals of white ethnicity in the development dataset (74.8% vs 35.4%). The development dataset had fewer people in the lower quintiles of deprivation and fewer with a diagnosis of schizophrenia (14.6% vs 22.5%). In the development dataset, individuals had a lower number of psychiatric admissions prior to discharge from EIP and had a shorter duration of care from EIP services (a mean of 570.9 vs 846.0). More individuals were discharged to primary care in the development dataset (83.6% vs 74.2%).

**Table 1:** Demographics, clinical service use and outcome comparison between Oxford Health NHS Foundation Trust and West London NHS Trust.

	Oxford Health (*n* = 831)		West London (*n* = 1393)	
Variable	Missing no. (%)	Mean (SD) no. (%)	Missing no. (%)	Mean (SD) no. (%)
Gender, female	1 (0.12%)	307 (37%)	0 (0%)	516 (37%)
Age	0 (0%)	25.6 (7.6)	0 (0%)	26.7 (5.2)
Ethnicity, White	128 (15.4%)	526 (74.8%)	25 (1.8%)	485 (35.4%)
Deprivation index, quintile^a^	46 (5.5%)		54 (3.9%)	
1		72 (9.2%)		342 (25.5%)
2		157 (20.0%)		566 (42.3%)
3		167 (21.3%)		283 (21.1%)
4		151 (19.2%)		120 (8.9%)
5		236 (30.1)		28 (2.1%)
Diagnosis, schizophrenia	5 (0.6%)	121 (14.6%)	77 (5.53%)	296 (22.5%)
Substance misuse, yes	0 (0%)	157 (18.9%)	0 (0%)	228 (16.4%)
Number of admissions prior to discharge	0 (0%)	–	0 (0%)	–
0	–	499 (60.0%)	–	717 (51.5%)
1	–	230 (27.7%)	–	345 (25.4%)
2–4	–	96 (11.6%)	–	295 (21.2%)
5 or more	–	6 (0.7%)	–	28 (2.0%)
Length of EIP, days	0 (0%)	570.9 (421.3)	0 (0%)	846 (497.1)
Discharge destination, primary care	0 (0%)	695 (83.6%)	0 (0%)	1034 (74.2%)
Outcome, admitted within 12 months of discharge	0 (0%)	79 (9.5%)	0 (0%)	162 (11.6%)

^a^Lower decile equals more deprived.

### Model development

In the development dataset, 79 (9.5%) individuals were admitted to a psychiatric hospital within 12 months of being discharged from EIP services. Being male was associated with lower odds of admission (OR = 0.562, 95% CI 0.328: 0.963, *p* = 0.036). A diagnosis of schizophrenia (OR = 2.571, 95% CI 1.433; 4.613, *p* = 0.002), a higher number of previous admissions (1.294, 95% CI 1.037; 1.613, *p* = 0.022), and a diagnosis of substance misuse (3.242, 95% CI = 1.815; 5.788, *p* < 0.001) were all associated with higher odds of readmission (Table 2).

**Table 2:** Logistic model performance of the development dataset.

Predictor	OR	95% CI	*p*-value	Change in C-index^b^, if removed^c^
Gender, male	0.562	0.328; 0.963	0.036	−0.021
Age	0.998	0.963; 1.033	0.901	0.006
Ethnicity, white	1.300	0.687; 2.461	0.419	0.002
LSOA^a^	0.973	0.880; 1.076	0.594	0.003
Diagnosis, schizophrenia	2.571	1.433; 4.613	0.002	−0.006
Duration of EIP, days	1.001	1.000; 1.001	0.088	−0.001
Number of admissions prior to EIP discharge	1.294	1.037; 1.613	0.022	−0.014
Substance misuse diagnosis	3.242	1.815; 5.788	<0.001	−0.057

^a^Lower super output area, a measure of local area deprivation.

^b^Harrell’s C-index (equivalent to the area under the receiver operating characteristic curve).

^c^Removed from the full model with all predictors included.

**Fig. 1:**
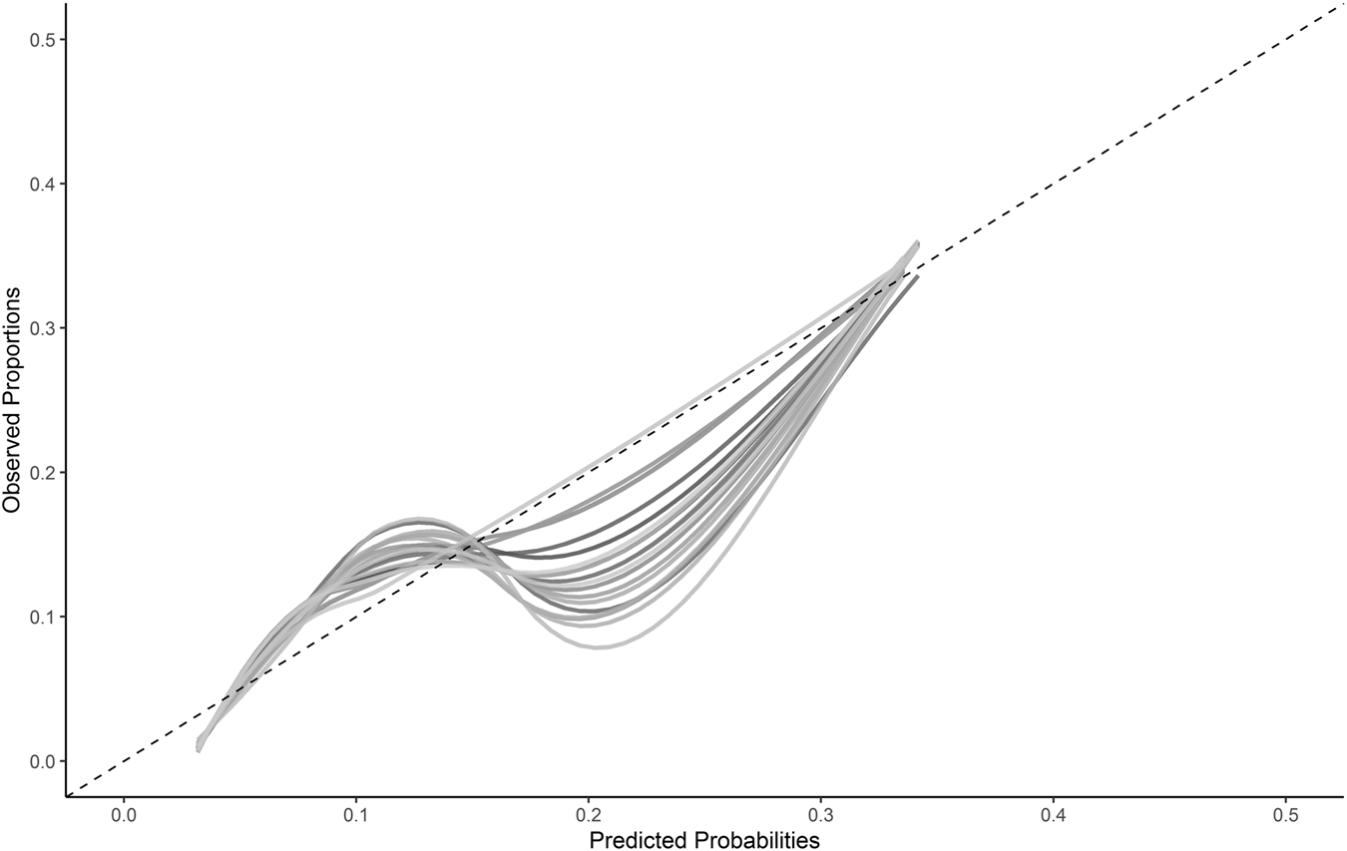
Calibration plot of development dataset. Fitted lines are the model predicted probabilities categorized into 10 groups for each of the 20 imputed datasets.

Overall performance measured with the Brier Score was 0.078. Internal bootstrap corrected discrimination showed fair performance with a C-statistic of 0.76 (95% CI 0.75; 0.77). The model showed slight overfitting with calibration-in-the-large of less than 0.01 (95% CI −0.25; 0.24); and a bootstrap corrected calibration slope of 0.89 (95% CI 0.88; 0.89). Calibration plots of the imputed datasets showed an underestimation at lower predicted probabilities and an overestimation at higher predicted probabilities, which is suggestive of overfitting (Fig. 1 and Supplementary Fig. 2). Table 2 provides further information on the contribution of individual predictor variables to overall discriminative performance. Predictors associated with the largest decreases in the C-index when removed from the model were substance misuse, gender, and the number of admissions to hospital.

Conducting a sensitivity analysis using only complete cases showed that OR point estimates were similar to the imputed model but had wider confidence intervals (Supplementary Table 2). Being male was no longer significantly associated with lower odds of admission (OR = 0.561, 95% CI 0.314: 1.001, *p* = 0.050) nor was the number of previous admissions (OR = 1.246, 95% CI 0.989; 1.571, *p* = 0.062). Discrimination (C-statistic = 0.77, 95% CI 0.72; 0.82), calibration in the large (<0.01, 95% CI −0.27; 0.25) showed a similar trend of comparable point estimates but with wider confidence intervals, while overall performance measured with the Brier Score was slightly worse at 0.085.

### External validation

**Fig. 2:**
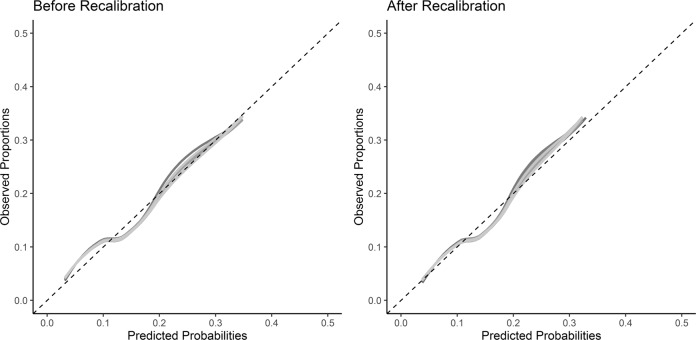
Calibration plot of the validation dataset before (left) and after (right) recalibration. Fitted lines are the model predicted probabilities categorized into 10 groups for each of the 20 imputed datasets.

We applied the coefficients derived in the development model (Supplementary Table 3) to individuals in the external validation dataset. Discrimination was fair in the validation dataset with a C-statistic = 0.70 (95% CI 0.66; 0.75). The Brier score was 0.094. There was some miscalibration, with calibration-in-the-large 0.06 (95% CI −0.12; 0.23) and a calibration slope of 0.86 (95% CI 0.68; 1.05). Inspection of the calibration plots showed a slight underestimation of prediction estimates at the lower probability range (0.05–0.1) and overestimation at the low-to-mid probability range (0.1–0.2, Fig. 2 and Supplementary Fig. 3).

Recalibration using the intercept and calibration slope as an adjustment factor resulted in a slightly different calibration curve, with calibration-in-the-large of less than −0.01 (95% CI −0.17; 0.167) and a calibration slope of 1 (95% CI 0.78; 1.22). Calibration plots showed closer estimation to the reference line (Fig. 2 and Supplementary Fig. 3) but with slight underestimation of risk at the higher predicted probabilities. The c-statistic remained the same (C-statistic = 0.70, 95% CI 0.66; 0.75), and the Brier score (0.094) improved slightly.

**Fig. 3:**
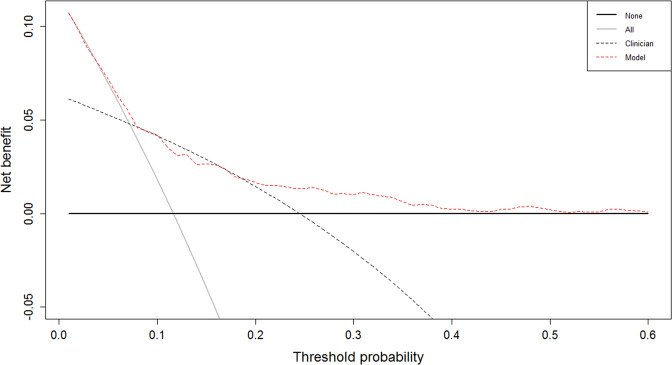
Decision curve analysis. Black horizontal line represents treating none, the grey diagonal line represents treating all, the black dotted line represents the actual discharge destination (i.e. transfer to secondary care), the red dotted line represents the prediction model.

Our decision curve analysis showed a net-benefit for use of the prediction model over standard discharge outcomes for the prediction of admission to hospital for a range of threshold probabilities between 20% and 40% (i.e. when the threshold probability to treat or not to treat is higher, Fig. 3).

## Discussion

This is the first externally validated prognostic model of readmission to hospital after discharge from EIP services. It used routinely recorded EHR data to predict readmission to a psychiatric hospital within 12 months of discharge from EIP services. The development and external validation discrimination performance (AUC 0.76 and AUC 0.70, respectively) were similar to risk prediction models already used in routine clinical practice such as cancer and cardiovascular disease^28^, and performed better than published prognostic models of acute psychiatric hospital readmission (ranging from AUC 0.59 to AUC 0.63)^25^ and acute hospital readmissions (AUC 0.50–AUC 0.83)^41^. A decision curve analysis showed that our model produces a net-benefit above usual discharge outcomes when the threshold probability was higher.

Our external validation dataset case-mix was different for a number of demographic and clinical characteristics; individuals in the validation dataset were older, there was more ethnic diversity, higher deprivation, more had a diagnosis of schizophrenia and a greater number of admissions to inpatient hospitals prior to their discharge from EIP, they had more days under the care of the EIP team, and fewer were discharged to primary care. These clinical differences are all indicative of a more severely ill population, although this did not result in significantly more admissions to hospital after discharge. In the development model, there was evidence from the calibration plots of overfitting in the model. Examining the validation model calibration suggested there was evidence of both over and underestimation of predicted probabilities. Recalibration of the intercept and model coefficients partly corrected this at the lower probabilities but created underestimation at the high probabilities. Underestimation at higher predicted probabilities may be more desirable as those persons are likely to be considered as suitable for secondary services by standard clinical decision-making processes, while those at lower probability are likely to be edge cases where suitability for transfer to secondary care is more uncertain.

### Strengths and limitations

Our study has a number of strengths. It has demonstrated that it is possible to use easily gathered measures to produce a reasonably accurate model of readmission risk within 12 months of EIP discharge. All the predictors in our model are routinely available making it clinically feasible and practical. The tool could be populated automatically from EHRs and integrated within clinical information systems, enhancing the likelihood that the tool is used^30^.

There are a plethora of prognostic models within healthcare settings, but most model development and validation is poorly conducted and poorly reported^28^. We followed a strict protocol and guidance set out according to the TRIPOD statement^39^. We imputed missing data, used robust internal validation through bootstrapping, and evaluated model performance on an external dataset. We publish our model coefficients in full (Supplementary Table 2) and evaluated both discrimination and calibration. Discrimination and calibration were similar for both development and validation datasets, with an expected slight drop in performance in a validation dataset likely due to a largely different case-mix. A limitation of this model is that our confidence intervals in the logistic regression were wide. This may be due to only having few admission events in the derivation dataset, and the derivation sample was likely underpowered for the analysis^42^. Given this likelihood of overfitting, a priority for this prognostic model could be updating the model intercept and coefficients in a larger dataset with more events.

We externally validated our model in a sample with a largely different case-mix and there was only a slight drop in its performance. It is therefore likely to be generalisable, at least to other EIP services in England. Our samples were recruited from the entire population of clinical EIP services rather than a research sample so we believe it is representative of the general EIP population in England although we were missing data on individuals who moved out of area. Further refinement of the model, using wider sources of data with consideration of other clinically important but routinely collect variables, such as familial psychiatric history, could improve performance. Data collection from larger samples with more events would enable more complex models to be constructed in later iterations similar to the updating used in other prognostic models such as QRISK^17^. Another limitation is that our method of multiply imputing data and then bootstrapping the multiply imputed data may have produced optimistic estimates in comparison to alternative methods such as multiple imputations of the bootstrapped samples^43^. Both development and validation datasets were conducted within the context of NHS EIP services and the transportability to international EIP settings is also unknown and further validation is required.

We used ethnicity as a parameter in our model. While it did not make a considerable change to predictive discrimination (Table 2), the use of ethnicity within our model needs careful consideration. The incidence of psychosis is higher in minority ethnic groups in Western countries^44^, while long-term outcomes in those with psychosis is worse in some minority ethnic groups^45^. There is also considerable evidence of systematic bias towards people of non-white ethnicity in psychiatric services, such as people from Black African or Black Caribbean ethnicities being more likely to be involuntarily admitted to hospital^46^. The inclusion or exclusion of a parameter in which the indicator may lend itself to the outcome, or mirror human bias (i.e. the model’s use of ethnicity as a proxy for racial bias towards the increased admission of people from minority ethnic backgrounds), needs further testing within the model, but also with how the model is used within psychiatric services. We believe that this is another reason why transparent reporting of prediction models (e.g. reporting model coefficients) is important when using parameters such as ethnicity, rather than the use of models with inaccessible algorithms hidden within prediction tools and machine learning algorithms, as this will encourage examination of the influence of these parameters on model decisions.

### Use of the model in clinical practice

Another important consideration for this prognostic tool is how it is used. Clinicians frequently use prognostic decision making to guide investigations and treatment. They traditionally make these decisions using their clinical judgement—a blend of prior experience and known evidence of risk factors and outcomes. However, in medicine and in particular psychiatry, decisions are often probabilistic because the information we gather is imperfect and outcomes are often uncertain^47,48^. The use of a prognostic tool modelled at a static point in time at the population level to make binary decisions about treatment for an individual in a dynamic treatment environment, for instance, whether to discharge them to primary care or refer them to further secondary care, is unlikely to prove accurate or useful^49^.

Our prediction model may be more useful when used as an adjunct to clinical decision making at the end of EIP treatment^50^. We believe it has a number of potential uses. Firstly, it could be used directly with the individual as to inform discussions between clinician and that individual about the preference for continued specialist mental health care treatment. The transition from EIP to the next stage of treatment, whether that be treatment by a primary care provider or by a different mental health team is a critical moment in an individual’s journey following treatment for psychosis. Individuals can have conflicting feelings about their needs for further treatment, and their readiness for the inevitable change in the care they receive, thus clinicians’ communication and planning with individuals about their discharge can be influential in smoothing this process^6–8^. Sensitive use of the tool could act in discussions as an indicator of future need for care.

Second, it could be used to make more accurate decisions around the allocation of secondary care resources. Both community mental health and inpatient care incur high economic costs. Giving secondary care to all individuals treated by EIP is unlikely to be economically viable, and not all individuals want or need the type of care provided by community mental health teams^8^. This tool could be used to better target those most likely to relapse, and therefore those most likely to derive the benefit from secondary care, as these are the people for whom the resource will deliver the greatest absolute reduction in risk of relapse.

Third, it could be used within health services during transition periods to communicate risk to other agencies. Access to further care after EIP is often difficult due to differences in eligibility and assessment criteria^7^. Clear measurement of individual risk could be used by EIP teams to communicate significant risk—that of hospitalisation—to other secondary care agencies at the stage of referral.

All of these potential benefits need to be further explored. Future work on the acceptability and feasibility of the tool for individuals with psychosis and the clinicians who will be using it is vital. For those with psychosis, we need to find out whether the communication of this predicted risk is helpful or wanted, and if so, how to communicate it accurately and sensitively. For clinicians, we need to ascertain whether they can use the tool, and how best to do so. We also need to study how its use influences outcomes in terms of the number of referrals to adult community health teams, reduction in readmissions, or improvement in clinician–patient communication and satisfaction. Paramount to this would be the use of co-production during the further development and implementation of the mode with clinicians and service users to increase acceptability and support its use in a shared decision-making process.

## Supplementary information

Supplementary materials

## Data Availability

Data are available upon reasonable request from the corresponding author. The data are not publically available as they are pseudonymised medical records. All data remain within the UK CRIS platform and requests for data can be made to the relevant CRIS administrator at each Trust.
