# Supplementary material for: Development and external validation of an admission risk prediction model after treatment from early intervention in psychosis services
Source: Transl Psychiatry. 2021 Jan 11;11:35. doi: 10.1038/s41398-020-01172-y (PMC7801610; doi:10.1038/s41398-020-01172-y)
Supplement: Supplementary file 1 — Supplementary materials [file 41398_2020_1172_MOESM1_ESM.docx]

# Supplementary Materials

Supplementary table 1: TRIPOD Checklist: Prediction Model Development and Validation

| **Section/Topic** | **Item** |  | **Checklist Item** | **Page** |
| --- | --- | --- | --- | --- |
| **Title and abstract** | | | | |
| Title | 1 | D;V | Identify the study as developing and/or validating a multivariable prediction model, the target population, and the outcome to be predicted. | 1 |
| Abstract | 2 | D;V | Provide a summary of objectives, study design, setting, participants, sample size, predictors, outcome, statistical analysis, results, and conclusions. | 2 |
| **Introduction** | | | | |
| Background and objectives | 3a | D;V | - Explain the medical context (including whether diagnostic or prognostic) and rationale for developing or validating the multivariable prediction model, including references to existing models. | 3,4 |
|  | 3b | D;V | - Specify the objectives, including whether the study describes the development or validation of the model or both. | 4 |
| **Methods** | | | | |
| Source of data | 4a | D;V | - Describe the study design or source of data (e.g., randomized trial, cohort, or registry data), separately for the development and validation data sets, if applicable. | 4 |
|  | 4b | D;V | - Specify the key study dates, including start of accrual; end of accrual; and, if applicable, end of follow-up. | 5,6 |
| Participants | 5a | D;V | - Specify key elements of the study setting (e.g., primary care, secondary care, general population) including number and location of centres. | 4,5,6 |
|  | 5b | D;V | - Describe eligibility criteria for participants. | 5,6 |
|  | 5c | D;V | - Give details of treatments received, if relevant. | n/a |
| Outcome | 6a | D;V | - Clearly define the outcome that is predicted by the prediction model, including how and when assessed. | 5 |
|  | 6b | D;V | - Report any actions to blind assessment of the outcome to be predicted. | n/a |
| Predictors | 7a | D;V | - Clearly define all predictors used in developing or validating the multivariable prediction model, including how and when they were measured. | 5 |
|  | 7b | D;V | - Report any actions to blind assessment of predictors for the outcome and other predictors. | n/a |
| Sample size | 8 | D;V | - Explain how the study size was arrived at. | 4 |
| Missing data | 9 | D;V | - Describe how missing data were handled (e.g., complete-case analysis, single imputation, multiple imputation) with details of any imputation method. | 5,6 |
| Statistical analysis methods | 10a | D | - Describe how predictors were handled in the analyses. | 5,6 |
|  | 10b | D | - Specify type of model, all model-building procedures (including any predictor selection), and method for internal validation. | 5,6 |
|  | 10c | V | - For validation, describe how the predictions were calculated. | 6 |
|  | 10d | D;V | - Specify all measures used to assess model performance and, if relevant, to compare multiple models. | 6 |
|  | 10e | V | - Describe any model updating (e.g., recalibration) arising from the validation, if done. | 6 |
| Risk groups | 11 | D;V | Provide details on how risk groups were created, if done. | n/a |
| Development vs. validation | 12 | V | For validation, identify any differences from the development data in setting, eligibility criteria, outcome, and predictors. | 6 |
| **Results** | | | | |
| Participants | 13a | D;V | - Describe the flow of participants through the study, including the number of participants with and without the outcome and, if applicable, a summary of the follow-up time. A diagram may be helpful. | SF1 |
|  | 13b | D;V | - Describe the characteristics of the participants (basic demographics, clinical features, available predictors), including the number of participants with missing data for predictors and outcome. | T1 |
|  | 13c | V | - For validation, show a comparison with the development data of the distribution of important variables (demographics, predictors and outcome). | T1 |
| Model development | 14a | D | - Specify the number of participants and outcome events in each analysis. | 7 |
|  | 14b | D | - If done, report the unadjusted association between each candidate predictor and outcome. | n/a |
| Model specification | 15a | D | - Present the full prediction model to allow predictions for individuals (i.e., all regression coefficients, and model intercept or baseline survival at a given time point). | n/a |
|  | 15b | D | - Explain how to the use the prediction model. | ST2 |
| Model performance | 16 | D;V | - Report performance measures (with CIs) for the prediction model. | 7 |
| Model-updating | 17 | V | If done, report the results from any model updating (i.e., model specification, model performance). | 7 |
|  | | | | |
| Limitations | 18 | D;V | Discuss any limitations of the study (such as nonrepresentative sample, few events per predictor, missing data). | 8 |
| Interpretation | 19a | V | - For validation, discuss the results with reference to performance in the development data, and any other validation data. | 8 |
|  | 19b | D;V | - Give an overall interpretation of the results, considering objectives, limitations, results from similar studies, and other relevant evidence. | 9 |
| Implications | 20 | D;V | Discuss the potential clinical use of the model and implications for future research. | 9 |
| **Other information** | | | | |
| Supplementary information | 21 | D;V | Provide information about the availability of supplementary resources, such as study protocol, Web calculator, and data sets. | SFs |
| Funding | 22 | D;V | Give the source of funding and the role of the funders for the present study. | 10 |

**Supplementary Figure 1:** Flow diagram of participant eligibility


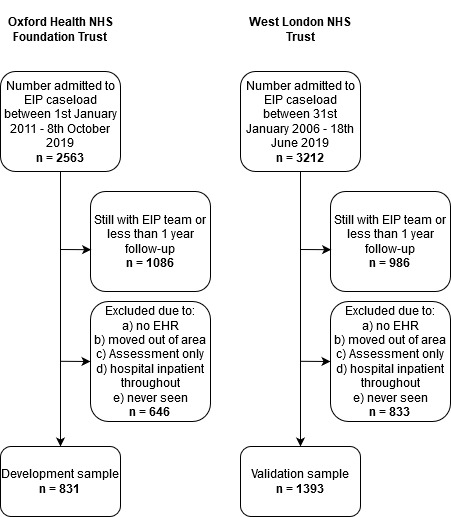


| **Supplementary Table 2: Complete case (n = 664) sensitivity analysis of logistic model performance of the development dataset** | | | |
| --- | --- | --- | --- |
| **Predictor** | **OR** | **95% CI** | **P-value** |
| **Gender**, *male* | 0.561 | 0.314; 1.001 | 0.050 |
| **Age** | 0.996 | 0.960; 1.033 | 0.821 |
| **Ethnicity**, *white* | 1.240 | 0.652; 2.359 | 0.512 |
| **LSOA**^1^ | 0.966 | 0.873; 1.069 | 0.504 |
| **Diagnosis**, *schizophrenia* | **2.537** | **1.364; 4.719** | **0.003** |
| **Duration of EIP**, *days* | 1.000 | 1.000; 1.001 | 0.405 |
| **Number of admissions prior to EIP discharge** | 1.246 | 0.989; 1.571 | 0.062 |
| **Substance misuse diagnosis** | **3.507** | **1.899; 6.478** | **< 0.001** |
| *1. Lower super output area, a measure of local area deprivation* | | | |

| **Supplementary Table 3: Bootstrap adjusted coefficients derived from logistic model** | |
| --- | --- |
| **Predictor** | **Coefficient** |
| **Intercept** | -3.311272473 |
| **Gender**, *male* | 0.576210412 |
| **Age** | -0.002234690 |
| **Ethnicity**, *white* | -0.262593105 |
| **LSOA** | -0.027267330 |
| **Diagnosis**, *schizophrenia* | 0.944245707 |
| **Duration of EIP**, *days* | 0.000525936 |
| **Number of admissions prior to EIP discharge** | 0.257462073 |
| **Substance misuse diagnosis** | 1.176060434 |


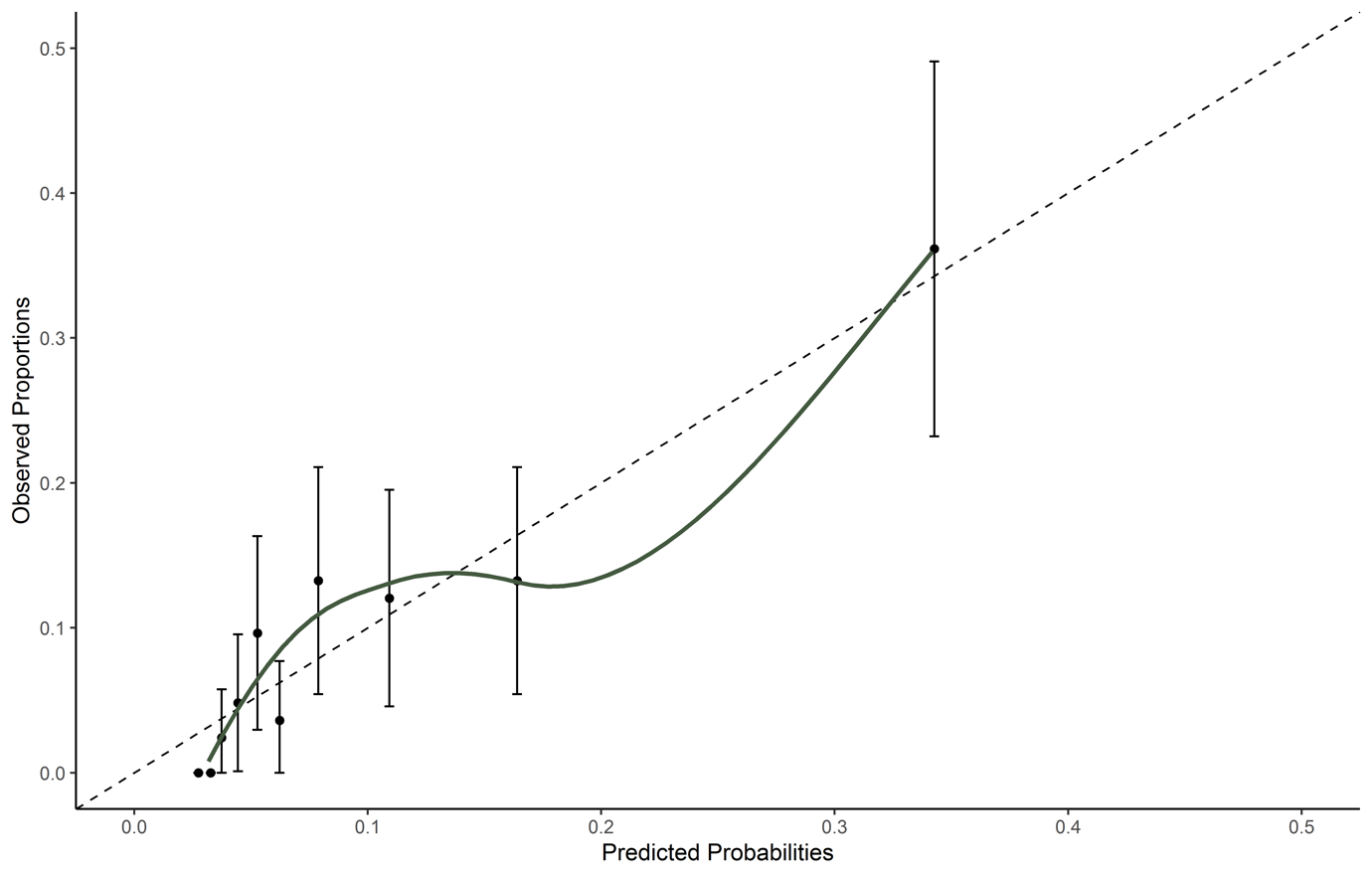


**Supplementary Figure 2.** Calibration plot of 1^st^ imputation of development dataset (as an example), showing fitted calibration lines across predicted probabilities categorized into 10 risk groups with confidence intervals.


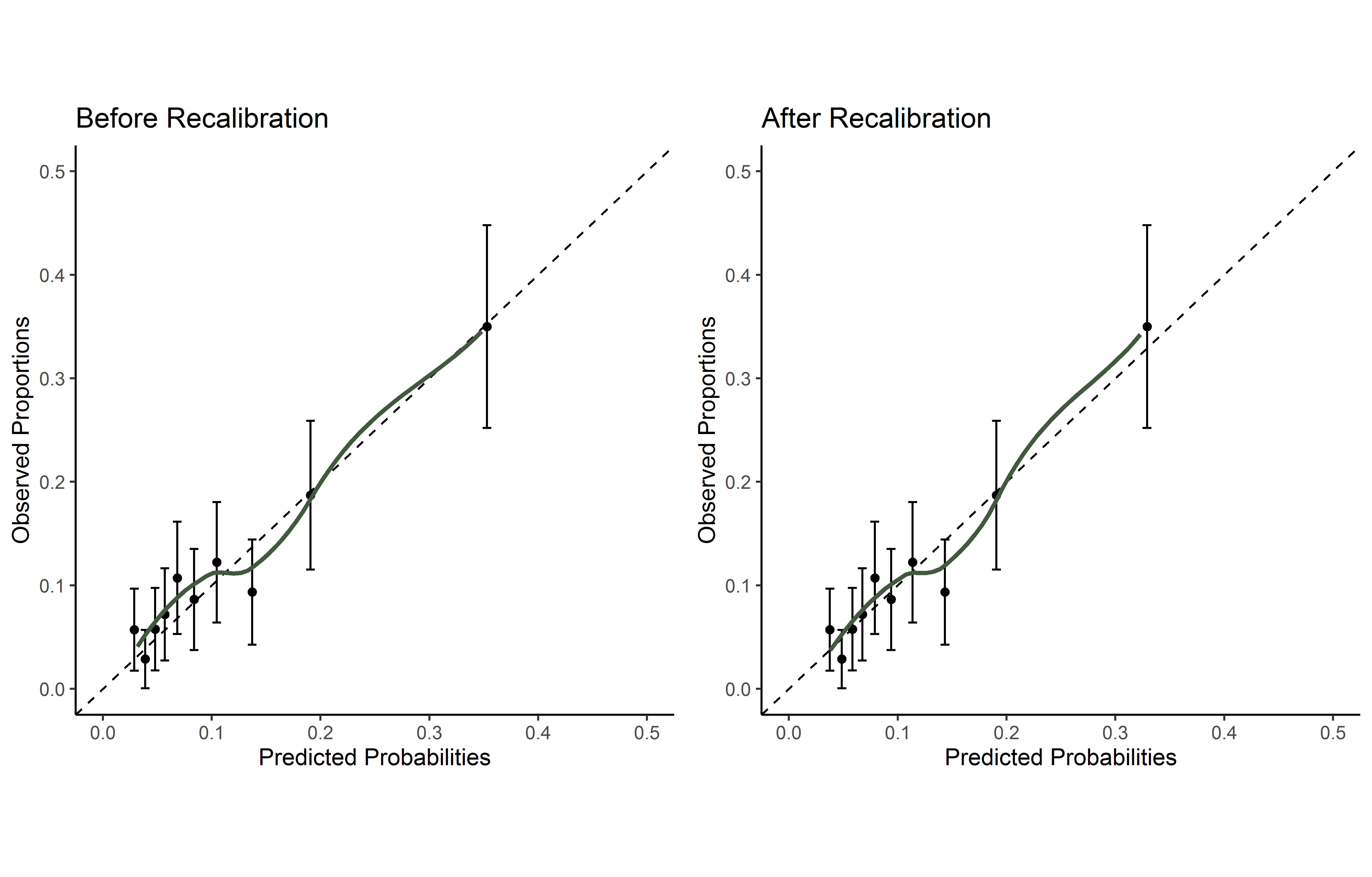


**Supplementary Figure 3.** Calibration plot of 1^st^ imputation of validation dataset (used as an example), before and after calibration showing fitted calibration lines across predicted probabilities categorized into 10 risk groups with confidence intervals.
